# The Phenotypic Spectrum of 15q13.3 Region Duplications: Report of 5 Patients

**DOI:** 10.3390/genes12071025

**Published:** 2021-07-01

**Authors:** Magdalena Budisteanu, Sorina Mihaela Papuc, Ioana Streata, Mihai Cucu, Andrei Pirvu, Simona Serban-Sosoi, Alina Erbescu, Emanuela Andrei, Catrinel Iliescu, Doina Ioana, Emilia Severin, Mihai Ioana, Aurora Arghir

**Affiliations:** 1 Department of Pediatric Neurology, Prof. Dr. Alex. Obregia Clinical Hospital of Psychiatry, 041914 Bucharest, Romania; magda_efrim@yahoo.com (M.B.); emaandrei@gmail.com (E.A.); catrinel.iliescu@gmail.com (C.I.); doinaioana70@gmail.com (D.I.); 2 Medical Genetics Laboratory, Victor Babes National Institute of Pathology, 050096 Bucharest, Romania; erbescua@gmail.com (A.E.); aurora.arghir@ivb.ro (A.A.); 3 Department of Genetics, Faculty of Medicine, Titu Maiorescu University, 031593 Bucharest, Romania; 4 Human Genomics Laboratory, Faculty of Medicine, University of Medicine and Pharmacy of Craiova, 200349 Craiova, Romania; ioana.streata@umfcv.ro (I.S.); mihai.cucu@umfcv.ro (M.C.); andrei.crgm@gmail.com (A.P.); simona.sosoi@umfcv.ro (S.S.-S.); mihai.ioana@umfcv.ro (M.I.); 5 Regional Center of Medical Genetics Dolj, 200642 Craiova, Romania; 6 Department of Genetics, Faculty of Dental Medicine, “Carol Davila” University of Medicine and Pharmacy Bucharest, 050474 Bucharest, Romania; emilia.severin@umfcd.ro

**Keywords:** ASDs, clinical significance, phenotype variability, CHRNA7 duplications, chromosomal microarrays

## Abstract

Chromosome 15q13.3 microduplications are associated with a wide spectrum of clinical presentations ranging from normal to different neuropsychiatric conditions, such as developmental delay (DD), intellectual disability (ID), epilepsy, hypotonia, autism spectrum disorders (ASD), attention-deficit hyperactivity disorder, and schizophrenia. The smallest region of overlap for 15q13.3 duplications encompasses the Cholinergic Receptor Nicotinic Alpha 7 Subunit (*CHRNA7*) gene, a strong candidate for the behavioral abnormalities. We report on a series of five patients with 15q13.3 duplications detected by chromosomal microarray. The size of the duplications ranged from 378 to 537 kb, and involved the *CHRNA7* gene in all patients. The most common clinical features, present in all patients, were speech delay, autistic behavior, and muscle hypotonia; DD/ID was present in three patients. One patient presented epileptic seizures; EEG anomalies were observed in three patients. No consistent dysmorphic features were noted. Neuroimaging studies revealed anomalies in two patients: Dandy–Walker malformation and a right temporal cyst. 15q13.3 duplications are associated with various neuropsychiatric features, including speech delay, hypotonia, ASD, and ID, also present in our patient group. Our study brings detailed clinical and molecular data from five ASD patients with 15q13.3 microduplications involving the *CHRNA7* gene, contributing to the existing knowledge about the association of 15q13.3 duplications with neuropsychiatric phenotypes.

## 1. Introduction

Copy number variations (CNVs), defined as duplications or deletions of genomic regions typically over 1 kb in size, play important roles in evolution, human genetic variation and human disorders. CNVs have been involved in many neuropsychiatric disorders, including intellectual disability (ID)/developmental delay (DD), autism spectrum disorder (ASD), bipolar disorder (BD), and schizophrenia (SCZ). There is a significant overlap of CNVs across neuropsychiatric disorders [1,2]; one of the CNVs considered to be at the root of several neurodevelopmental disorders involves 15q13.3.

Chromosome 15q11–13 shows a high frequency of genomic rearrangements. The genomic instability is attributed to the presence of low copy repeats (LCRs) which mediate aberrant interchromosomal exchanges during meiosis by non-allelic homologous recombination (NAHR) [3].

Microdeletions and microduplications affecting 15q13.3 were reported with a highly variable phenotype, ranging from normal to different neurological manifestations [4,5,6]. The minimal critical region reported in affected patients encompasses the *CHRNA7* (Cholinergic receptor nicotinic alpha 7 subunit) gene, proposed as candidate for the neuropsychiatric phenotype [7]. Sharp et al. (2008) reported on nine patients with 15q13.3 deletions and ID, epilepsy and mild dysmorphic features and proposed a novel clinical entity [4], Chromosome 15q13.3 microdeletion syndrome (MIM # 612001) [8]. A description of several series of patients with 15q13.3 interstitial deletions, revealed a wide range of neuropsychiatric phenotypes including ID/DD, speech problems, seizures, ASD, BD and SCZ [4,5,9,10]. While patients with 15q13.3 heterozygous deletions present highly variable clinical manifestations—even in the affected members of the same family—patients with homozygous deletions are more severely affected, presenting epileptic encephalopathy, hypotonia, and growth retardation [11,12,13].

Small microduplications of 15q13.3, involving mainly the *CHRNA7* gene, have been frequently detected in microarray studies. *CHRNA7* duplications have been associated with a large spectrum of neurodevelopmental disorders, including ID/DD, language development disorders, ASD, mood disorders, attention deficit hyperactivity disorder (ADHD), childhood-onset schizophrenia (COS), Tourette syndrome (TS), obsessive compulsive disorder, and epilepsy [5,9,14,15,16,17,18,19,20,21]. Triplications of 15q.13.3 involving the *CHRNA7* gene were also reported in patients with cognitive impairment and neuropsychiatric phenotypes [22]. However, 15q13.3 microduplications are equally prevalent in clinical cases and in the general population [6,23,24,25], making their contribution to pathogenicity difficult to evaluate.

Several studies reported neuropsychiatric phenotypes in 15q13.3 microduplication; however, the number of affected patients with a complete clinical description available in the literature is still low. Van Bon et al. (2009) reported on four patients with 15q13.1–13.3 microduplication; their main symptoms were ID, psychiatric disorders (ASD, disruptive behaviors), hypotonia, obesity, and recurrent ear infections [9]. Szafranski et al. (2010) investigated 55 patients with small microduplications involving *CHRNA7;* however, complete clinical descriptions were available only for 11 patients and included DD/ID, muscular hypotonia, and a variety of neuropsychiatric disorders (ASD, ADHD, BD, anxiety, and severe pica) [14]. Another study presented a child with 15q13.3 microduplication and speech delay, ASD, seizures, and brain malformations [18]. A total of 135 ASD patients were evaluated for the presence of *CHRNA7* rare variants as a risk factor for ASD in another study [19]. *CHRNA7* duplication was identified in only one patient and proved to be inherited from an unaffected father; the clinical description of the patient included ASD, moderate cognitive impairment, and complex partial seizures with secondary generalization. In 2016, Zhou et al. identified *CHRNA7* duplication in two patients with COS, while other carrier family members presented TS and dyslexia [21]. Gillentine et al. (2017) studied the clinical and behavioral phenotypes of 18 children with duplications involving the *CHRNA7* gene and noted that these duplications exhibit variable expressivity and incomplete penetrance (the inheritance of the CNV from an unaffected parent occurred in 35% of the cases; the same duplication was also observed in a healthy sibling) [6]. Variable expressivity referred to ID severity ranging from borderline to severe, additional diagnoses of ASD (30%) and ADHD (40%) as well as variable ADI-R and ADOS-2 scores among the patients with behavioral problems. Obsessive–compulsive and aggressive behaviors (28%), bipolar disorder and/or anxiety (17%) were also included in the clinical findings.

Although both microdeletion and microduplication of 15q13.3 share the same spectrum of neuropsychiatric disorders, microduplications tend to have a higher degree of variability in expressivity and penetrance making its detection and clinical interpretation of these anomalies more difficult. In addition, compared with microdeletions, microduplications are more often inherited than de novo, adding to the challenges of clinical interpretation and management [15].

The pathogenic contribution of small 15q13.3 microduplications in various neuropsychiatric disorders is currently under debate. To better understand the complex genotype-phenotype correlations of these microduplications, detailed clinical characterization of larger study groups of individuals carrying these CNVs would be useful, especially with longitudinal follow-up. With this aim, the current study presents five new cases with 15q13.3 microduplications involving the *CHRNA7* gene and associated neuropsychiatric conditions.

## 2. Materials and Methods

The patients were referred to our Pediatric Neurology and Child and Adolescent Psychiatry Departments for clinical evaluation of neurodevelopmental disorders. Genetic testing was performed in a clinical setting for two patients (patients 1 and 2) for developmental delay. Three patients were enrolled in a research project focused on the genetics, clinical and neuroimaging aspects of ASD (patients 3–5). Personal history, psychomotor development, behavior, and presence of dysmorphic features were investigated and recorded for all patients. Psychological tests were adapted to the age of the patients and included the Portage test, Raven and WISC. Neuroimaging and EEG studies were performed for four patients and three patients, respectively. EEG studies were performed using a 32-channel electroencephalograph according to the standard protocol: wake-up recording with closed eyes and open eyes—5 min; hyperventilation stimulation—5 min; intermittent photic stimulation. Brain MRI tests were performed using a 1.5 Tesla system, according to the standard protocol. High-resolution array based comparative genomic hybridization (array-CGH) was performed for all patients using Agilent SurePrint G3 Human CGH Microarray Kits (Agilent Technologies, Santa Clara, CA, USA). Two different platforms, 8 x 60K ISCA v2 with an overall median probe spacing of 60 kb (patients 1, 2) and 4 x 180K with an overall median probe spacing of 13 kb (patients 3, 4, 5) were used, according to previously described protocols [26,27]. The analysis was not performed in the parents of the affected subjects as they, for various reasons, were not available.

Ethical approval for the study was obtained from the Ethics Committee of Prof Dr Alex Obregia Clinical Hospital of Psychiatry (Protocol approval No. 33/26.11.2019 and 134/2013). A written informed consent was signed by all patients’ parents or legal guardians.

## 3. Results

Five patients, four boys and one girl, two of them siblings (patients 4 and 5), aged between 26 months and 15 years, were included in the study. The clinical features of all patients are summarized in Table 1. The clinical features common to all five patients were speech delay, autistic behavior, and muscle hypotonia. One patient presented focal epileptic seizures; EEG anomalies were observed in three cases and were represented by focal discharges of spike-and-wave complexes and spikes. No consistent dysmorphic features were noted in our patients, and growth parameters (i.e., height, weight, and head circumference) were in the normal range in all cases. Neuroimaging studies identified anomalies in two patients: a right temporal cyst (patient 1) and Dandy–Walker malformation (patient 2).

We observed clinical variability in our patients regarding the achievement of motor milestones, ranging from mild (patient 3) and moderate (patient 2) to severe motor delay (patient 1; at 9 years of age, he cannot walk independently). Patient 2 was born prematurely after 27 weeks of gestation. Patient 1 presented a large right temporal cyst, and surgical intervention in early childhood led to secondary left hemiparesis. We considered the delay in motor development in patients 1 and 2 related mostly to these described factors. Impairment of fine motor skills and muscle hypotonia were present in all patients.

Speech development was also impaired in our patients, a delay in achieving the first words and sentences being noted for the entire group. The degree of speech delay was mild for patient 5 (first words at 15 months, first simple sentences at 2 years and 7 months), moderate for patients 2, 4 (first words after 3 years) and 3 (no words at 2 years and 4 months), and severe for patient 1 (first words at 4 years and 6 months, first sentences at 6 years). Three of the four patients with mild to moderate language delay (1, 2 and 4) had dyslalia, with inability to properly articulate some words.

Regarding cognitive development, three patients had developmental delay (DD)/intellectual disability (ID) and two patients (4 and 5) had normal cognitive development. Patients 2 and 3 had moderate DD/ID. Patient 1 had a variable evolution with mild DD (DQ 51) at the time of first presentation (26 months) followed by a decrease of IQ (45 at 9 years) after the neurosurgical intervention due to a large right temporal cyst.

A variety of behavior problems were noted in our patients: hyperkinesia (patients 2 and 4), aggressive behavior (patients 1 and 2), low frustration threshold (patients 1, 2, 5), stereotypic movements (patients 1, 2 and 3), poor social interaction skills (all patients), sleep problems (patient 3) and anxiety disorder (patient 5).

Psychiatric examination revealed autistic behavior in all patients, according to the DSM 5 criteria. In patients 1 and 3, domains A and B were equally affected, in patient 2 domain B was more affected, and in patients 4 and 5, domain A was more affected. ADOS (performed for three patients) and ADI-R (performed in two patients) tests confirmed the clinical diagnosis of ASD in all cases.

**Figure 1:**
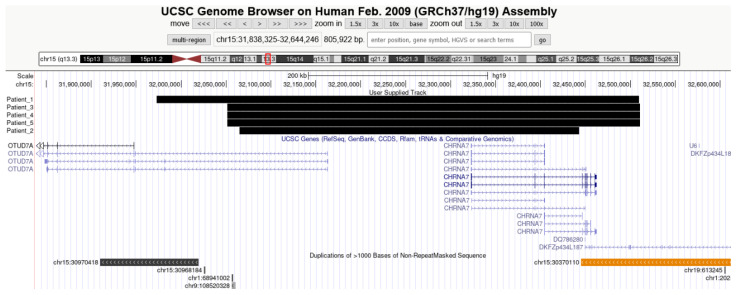
Copy number variants at 15q13.3 region: the black bars illustrate the duplications found in our patients, involving CHRNA7 and OTUD7A genes (UCSC genes track), flanked by LCR elements (Segmental Duplication track). (UCSC Genome Browser; https://genome.ucsc.edu/, accessed on 20 April 2021).

All patients had heterozygous duplications of 15q13.3, detected by array-CGH (Figure 1). The size of the duplications ranged from 378 to 537 kb, and included the *CHRNA7* and OTUD7A genes (Table 2).

## 4. Discussion

Genetic rearrangements involving 15q11–13 are well recognized for their association with neurological and neuropsychiatric phenotypes [4,5,9,10]. The high density of low copy repeats (LCRs) in this region prone to recurrent structural variation, such as (micro)deletions, (micro)duplications or complex genomic rearrangements of various sizes depending on the involved LCRs [7]. Six breakpoint subregions, BP1-BP6, were described. Besides, CNVs involving the Prader–Willi/Angelman Syndrome region, various other genomic imbalances, distal to BP3, were reported in patients with neurodevelopmental disorders [4]. These deletions and duplications vary in size and are preferentially located between BP3–BP5. Chromosome 15q13.3 deletions occur most frequently between BP4 and BP5, span ~1.5Mb, and encompass six coding genes and one microRNA (*FAN1*, *MTMR10*, *TRPM1*, *KLF13*, *OTUD7A*, *CHRNA7* and hsa-mir-211). Chromosome 15q13.3 duplications are frequently smaller (350–680 kb) and localized between Distal-CHRNA7-LCR (D-CHRNA7-LCR) and BP5 [7]. These duplications involve at least the *CHRNA7* gene (either the entire gene or part of it), with or without the first noncoding exon of the longest *OTUD7A* isoform. All our cases present duplications of 15q13.3 spanning less than 550 kb and encompass the *CHRNA7* gene (the entire gene in 4 patients and part of it in one patient). In addition, the first one or two non-coding exons and adjacent introns of the longest *OTUD7A* transcript were encompassed by the duplication.

As the minimal region of overlap for 15q13.3 microdeletions and microduplications encompasses *CHRNA7*, this gene has been proposed as the main candidate for the neuropsychiatric phenotypes in affected patients [7]. The *CHRNA7* gene encodes a member of the nicotinic acetylcholine receptors family (nAChRs), α7nAChRs. These receptors are ligand-gated ion channels with a pentameric structure of homologous subunits. They are endogenously stimulated by acetylcholine, leading to opening of ion channels and allowing the flux of the cations Na^+^, K^+^, and Ca^2+^ across the plasma membrane [28]. nAChRs are highly expressed in the human brain, with the highest level of α7nAChRs in hippocampus, a brain region known to be important for the cognitive processes. The homopentameric α7nAChRs have important functions such as mediation of signal transmission at synapse and modulation of neurotransmitter release [29].

The clinical significance of *CHRNA7* duplications is still under debate, due to the incomplete penetrance and variable expressivity of neuropsychiatric phenotypes. A wide spectrum of clinical features were reported in patients with 15q13.1–13.3 microduplication, including DD/ID, speech delay, seizures, hypotonia, brain malformations, and various psychiatric disorders (ASD, ADHD, COS, BD, obsessive–compulsive and aggressive behaviors, anxiety, severe pica) [6,9,14,18,19,21].

The results of several different case-control studies delineating genomic risk factors in neurodevelopmental and neuropsychiatric disorders were controversial with regard to *CHRNA7* duplication as potential risk factor. Helbig et al. (2009) evaluated the genomic risk factors for idiopathic generalized epilepsy (IGE) and found duplications involving *CHRNA7* in 0.98% of the patients (12 of 1223) and 0.62% of the controls (23 of 3699). The study concluded that 15q13.3 duplications are not major risk factors for IGE [30]. Coe et al. (2014) found no significant enrichment of small duplications involving *CHRNA7* in a cohort of pediatric patients with various developmental disorders compared with controls [24]. Similarly, Smajlagić et al. (2020) focused on population prevalence and inheritance pattern of recurrent CNVs associated with neurodevelopmental disorders finding that duplications involving only *CHRNA7* and/or *OTUD7A* genes are common, in comparison with deletions involving the same region [31]. The authors also suggested that these duplications are phenotypically neutral [31].

To better understand the contribution of *CHRNA7* CNVs to the clinical phenotype, patient-control studies and genotype–phenotype correlations were completed by animal models and induced pluripotent stem sells (iPSCs) studies. *Chrna7* knockout mice showed impaired working memory and attention span [32,33,34], but no behavioral or neuropsychiatric-like alterations were observed [35]. The effect of *CHRNA7* duplication has yet to be modeled in vivo. Thus, cellular models such as iPSCs-derived neural progenitor cells (NPCs) and organoids have the potential to overcome these limitations. Studies on iPSCs and NPCs derived from patients with heterozygous 15q13.3 deletions or duplications revealed a shared, potentially pathogenic mechanism for *CHRNA7* dosage alteration: a decrease in α7nAChR-dependent calcium flux [36,37].

Our study’s clinical findings are similar with those previously published [5,7,9,10,15,18,19,38]. Behavioral problems were frequently observed in our patients, all five patients having an ASD diagnosis. Social interaction and social communication impairments, the most prominent features, ranged from abnormal social approach to failure to engage in any form of social and emotionally-reciprocal interaction, and were associated with poorly integrated means of both verbal and non-verbal communication. These deficits were observed in all five patients. Additionally, three of our patients presented stereotyped motor movements, as well as repetitive patterns of behavior. This is also consistent with the clinical phenotype of 15q13.3 duplication described in previous studies [7,9,14].

Developmental delay was a common feature in our patients, speech being more frequently and more severely affected than motor development. All patients had speech delay, in terms of achieving first words and first sentences, and three of the four patients with developed language had language problems (dyslalia). This is considered the most common clinical feature in patients with 15q13.3 duplication. Only two patients had moderate to severe motor delay, but both of them had another problem that might explain this feature. These two patients also had moderate intellectual disability. Muscle hypotonia and fine motor inability were noted in all cases.

Epileptic seizures were noted in only one patient, EEG abnormalities were detected in three of the investigated patients, however. A reduced prevalence of epilepsy was also observed in other studies, epilepsy being less common in 15q13.3 duplication in comparison with 15q13.3 deletion [6].

Brain MRI was performed in four patients from our group. One patient presented a right temporal cyst, and another presented Dandy–Walker malformation. No specific brain anomalies were associated with 15q13.3 duplication in previous studies; most brain MRIs showed normal results. Several studies described various brain anomalies, such as neuronal migration disorder [18], left middle cranial fossa arachnoid cyst [22] and abnormal organization of the hippocampus [39].

Thus, ASD, language delay, and hypotonia were our patients’ core clinical features. ID, epilepsy, and motor development delay were observed in some, but not in all of our patients.

Study limitations include the small size of the patient group, absence of inheritance data and presence of other clinical conditions which could influence the clinical picture (such as the prematurity and the large temporal cyst).

## 5. Conclusions

Chromosome 15q13.3 microduplications involving the *CHRNA7* gene are frequently detected in microarray studies, both in clinical populations with developmental disorders and in healthy individuals. How these CNVs contribute to pathogenicity is currently debated, warranting further studies. The neuropsychiatric phenotypes described in patients with 15q13.3 microduplications include ASD, hypotonia, ID, and speech delay, all of which were present in our patient group.

Our study presents detailed clinical and molecular data from five new ASD patients with 15q13.3 duplications, involving the *CHRNA7* gene, contributing to the current knowledge about 15q13.3 duplications.

## Figures and Tables

**Table 1:** The clinical characteristics of the patients.

Pt No.	Gender	Age at Presentation	Dysmorphic Features	ID/DD (IQ/DQ)	Speech Delay	ASD	Feeding Difficulties	Hypotonia	Epilepsy	EEG Anomalies	Brain IRM Anomalies
1.	M	26 months → 9 years	−	+ (51 → 45)	+	+	−	+	−	+	+
2.	M	15 years	−	+ (40)	+	+	−	+	+	+	+
3.	M	2 yrs 4 months	−	+ (39)	+	+	−	+	−	NA	−
4.	F	4 years	−	− (73)	+	+	−	+	−	NA	NA
5.	M	10 years	−	− (90)	+	+	−	+	−	+	−

Abbreviations: + present; − absent; NA not available.

**Table 2:** Summary of the genomic characteristics of 15q13.3 duplicated regions in our patients.

Patient No.	Size	ISCN Formula	OMIM Genes
1.	537,280	arr[GRCh37] 15q13.3(31864691x2,31972646_32509926x3,32631629x2)	*OTUD7A*, *CHRNA7*
2.	378,087	arr[GRCh37] 15q13.3(31972706x2,32065000_32443078x3,32445199x2)	*OTUD7A*, *CHRNA7*
3.	459,630	arr[GRCh37] 15q13.3(32021793x2,32051233_32510863x3,32914080x2)	*OTUD7A*, *CHRNA7*
4.	459,630	arr[GRCh37] 15q13.3(32021793x2,32051233_32510863x3,32914080x2)	*OTUD7A*, *CHRNA7*
5.	459,630	arr[GRCh37] 15q13.3(32021793x2,32051233_32510863x3,32914080x2)	*OTUD7A*, *CHRNA7*

## Data Availability

The main data generated and analyzed in our study are included in this article.
